# SEROTONIN SYNDROME AFTER HIGH DOSAGE QUETIAPINE INTAKE: CASE REPORT AND BRIEF LITERATURE REVIEW

**DOI:** 10.1192/j.eurpsy.2023.1630

**Published:** 2023-07-19

**Authors:** P. Fountas, D. Kasimis, K. Goulas, N. Niamonitos, A. Basdekis, E. Thanasas, G. Charitakis, M. Koumpis

**Affiliations:** Psychiatry, Athens General Hospital Korgialenio-Benakio, Athens, Greece

## Abstract

**Introduction:**

Serotonin syndrome is a potentially life-threatening condition due to increased serotonergic activity in the CNS. Its most well-known causes are the ingestion of large amounts of serotonergic drugs or the inappropriate combination of two or more serotonergic antidepressants. Rarer causes of serotonin syndrome have been reported in the literature.

**Objectives:**

The aim of this paper is to highlight, through case presentation, rarer causes of serotonin syndrome.

**Methods:**

Case presentation (woman, 39 years old) who was hospitalized with clinical presentation of Serotonin syndrome, after pharmaceutical self-poisoning with a large amount of quetiapine (>20g). The search of the case data (clinical and paraclinical examination findings) was performed from the medical records and files of the 3rd Internal Medicine Clinic and the Liaison Psychiatry Department of our hospital. A literature search of similar cases (through PubMed) was performed.

**Results:**

A patient with a history of mood disorders, under treatment with venlafaxine, lamotrigine, quetiapine and aripiprazole, was admitted to the hospital ICU due to a decreased level of consciousness (GCS 11/15). Ingestion of approximately 100 tablets of Quetiapine 200mg was reported. The patient developed upper and lower limb myoclonus, supraventricular tachycardia (130bpm), nystagmus, bilateral mydriasis with normal photomotor reflexes, increased tendon reflexes, excessive sweating, upper limb muscle rigidity and abdominal flatulence with the presence of loud bowel sounds. Gastric lavage was performed and admission to the Internal Medicine department followed. Intravenous diazepam was administered, 30mg on the 1st 24 hour period and 20mg on the 2nd, with gradual tapering. From the very 2nd 24 hour period of hospitalization, her clinical condition showed significant improvement, with complete remission of symptoms and recovery of a satisfactory level of consciousness.

**Conclusions:**

Serotonin syndrome can be effectively treated if early recognition of symptoms is made. Clinicians should be particularly alert and suspect the possibility of serotonin syndrome in a patient with compatible symptoms by clinical examination, especially in case of overdose of a drug, with or without a serotonergic mechanism of action.

**Disclosure of Interest:**

None Declared

